# Cold Shock Proteins Are Expressed in the Retina Following Exposure to Low Temperatures

**DOI:** 10.1371/journal.pone.0161458

**Published:** 2016-08-24

**Authors:** Ignacio M. Larrayoz, Manuel Rey-Funes, Daniela S. Contartese, Federico Rolón, Anibal Sarotto, Veronica B. Dorfman, Cesar F. Loidl, Alfredo Martínez

**Affiliations:** 1 Angiogenesis Study Group, Center for Biomedical Research of La Rioja (CIBIR), 26006, Logroño, Spain; 2 Laboratorio de Neuropatología Experimental, Instituto de Biología Celular y Neurociencia “Prof. E. De Robertis” (IBCN), Facultad de Medicina, Universidad de Buenos Aires, CONICET, Paraguay 2155 (C1428ABG), Ciudad Autónoma de Buenos Aires, Argentina; 3 Centro de Estudios Biomédicos, Biotecnológicos, Ambientales y Diagnóstico (CEBBAD), Universidad Maimónides, Hidalgo 775 (C1405BCK), Ciudad Autónoma de Buenos Aires, Argentina; 4 Laboratorio de Neurociencia, Facultad de Ciencias Médicas, Universidad Católica de Cuyo. San Juan, Argentina; University of Florida, UNITED STATES

## Abstract

Hypothermia has been proposed as a therapeutic intervention for some retinal conditions, including ischemic insults. Cold exposure elevates expression of cold-shock proteins (CSP), including RNA-binding motif protein 3 (RBM3) and cold inducible RNA-binding protein (CIRP), but their presence in mammalian retina is so far unknown. Here we show the effects of hypothermia on the expression of these CSPs in retina-derived cell lines and in the retina of newborn and adult rats. Two cell lines of retinal origin, R28 and mRPE, were exposed to 32°C for different time periods and CSP expression was measured by qRT-PCR and Western blotting. Neonatal and adult Sprague-Dawley rats were exposed to a cold environment (8°C) and expression of CSPs in their retinas was studied by Western blotting, multiple inmunofluorescence, and confocal microscopy. RBM3 expression was upregulated by cold in both R28 and mRPE cells in a time-dependent fashion. On the other hand, CIRP was upregulated in R28 cells but not in mRPE. In vivo, expression of CSPs was negligible in the retina of newborn and adult rats kept at room temperature (24°C). Exposure to a cold environment elicited a strong expression of both proteins, especially in retinal pigment epithelium cells, photoreceptors, bipolar, amacrine and horizontal cells, Müller cells, and ganglion cells. In conclusion, CSP expression rapidly rises in the mammalian retina following exposure to hypothermia in a cell type-specific pattern. This observation may be at the basis of the molecular mechanism by which hypothermia exerts its therapeutic effects in the retina.

## Introduction

Hypothermia, or exposure to cold temperatures, is being used clinically as a therapeutical intervention to reduce the symptoms of several pathologies, including stroke [1–4], coronary artery bypass surgery [5], neurodegeneration after cardiopulmonary resuscitation [6], or neonatal asphyxia [7], among others. Although therapeutic hypothermia is well accepted for the clinical treatment of cardiological or central nervous system complications [8], at the moment it is not used in humans for diseases of the eye.

Nevertheless, a large number of animal studies has shown that hypothermia may be very useful in protecting the retina, especially in the context of ischemia [9–18]. Supporting these data, there are also some in vitro and ex vivo experiments showing that exposing isolated retinas or cultured retinal cells to cold temperatures results in reducing NMDA-mediated excitotoxicity [19], reducing secretion of vascular endothelial growth factor (VEGF) [20], and increasing survival against toxicants [21]. Hopefully, these promissing observations would be translated into clinical treatments soon.

Investigations in the last three decades have been trying to unravel the molecular mechanisms underlying the beneficial effects of hypothermia [22]. Usually, exposure to moderatelly cold temperatures induces a general reduction of metabolism and protein expression in all cells, but there is a small group of proteins, the so-termed cold-shock proteins (CSP), whose expression increases under these conditions. In mammals there are two main CSPs, RNA-binding motif protein 3 (RBM3) and cold inducible RNA-binding protein (CIRP or CIRBP). These proteins, which belong to the heterogeneous nuclear ribonucleoprotein family, bind to cellular RNAs and regulate their half-life and thus their expression potential and their final functions [23–26].

These CSPs have been identified in the mammalian brain [27–31] and CIRP was found in the brain and retina of a frog in the context of hybernation [32] but, to the best of our knowledge, they have not been localized in the mammalian retina. In this report, we identify the cold-induced responses of RBM3 and CIRP in cell lines of retinal origin as well as in retinas of neonatal and adult rats using quantitative real time PCR (qRT-PCR), Western blotting, and immunofluorescence.

## Materials and Methods

### 2.1. Cell lines and treatments

Two cell lines of retinal origin were used in this study: R28, which is an immortalized rat retinal precursor cell [33], and mRPE which is a retinal pigment epithelium cell line derived from a Rhesus monkey eye [34]. Both cell lines were a kind gift from Dr SP Becerra (National Eye Institute, NIH, Bethesda, MD). R28 cells were grown in DMEM medium supplemented with 10% fetal bovine serum (FBS). mRPE cells were grown in DMEM/F12 medium supplemented with 5% FBS, L-glutamine, Na-pyruvate, non-essential amino acids, and penicillin/streptomycin (all media and additives from Life Technologies, Alcobendas, Madrid, Spain). Cells in the exponential phase were incubated in an atmosphere containing 5% CO_2_ and 85% humidity. Temperature was modified as indicated.

### 2.2. Neonatal and adult rats

Male Sprague-Dawley albino rats with genetic quality and sanitary certification from the animal facility of our Institution were cared for in accordance with the guidelines published in the ARVO Statement for the Use of Animals in Ophthalmic and Vision Research, and in the NIH *Guide for the Care and Use of Laboratory Animals* (National Institutes of Health Publication No. 85–23, revised 1985, available from: Office of Science and Health Reports, National Center for Research Resources, Bethesda, MD 20892), and the principles presented in the "Guidelines for the Use of Animals in Neuroscience Research" by the Society for Neuroscience (available from the Society for Neuroscience, Washington, DC; published in Membership Directory of the Society, pp. xxvii-xxviii,1992). This study was approved by the Ethical Committee of CICUAL: “Comité Institucional para el Uso y Cuidado de Animales de Laboratorio” (Resolution N° 255/2014), Facultad de Medicina, Universidad de Buenos Aires, Argentina and by the US Department of Defense (ACURO, protocol number MR130239). Appropriate proceedings were performed to minimize the number of animals used and their suffering, pain, and discomfort. Animals were kept under standard laboratory conditions, with light/dark cycles of 12/12 h, and food and water were given *ad libitum*. Control animals were kept at 24°C whereas test animals were exposed to 8°C, in a cold room, for the indicated periods of time. Core temperature was measured with a digital thermometer (TES-1300, TES Electrical Electronic Corp. Taipei, Taiwan) connected to a K type thermocouple (TPK-01). Animals were sacrificed at the indicated times after exposure to cold temperatures.

### 2.3. RNA extraction and quantitative real time PCR

Rats were injected with a lethal dose of anesthetic (300 mg/Kg ketamine, Imalgene, Merial Laboratorios, Barcelona, Spain, + 30 mg/Kg xylazine, Xilagesic, Proyma Ganadera, Ciudad Real, Spain), the eyes were dissected out, and the anterior chamber and the lens were removed. Tissues or cell lines were homogenized with TRIzol (Invitrogen, Madrid, Spain) and RNA was isolated with RNeasy Mini kit including a DNAse I on-column digestion (Qiagen, Germantown, MD). One μg of total RNA was reverse-transcribed into first-strand cDNA using random primers and the SuperScript III kit (Invitrogen) in a total volume of 20 μL according to the manufacturer’s instructions. Reverse transcriptase was omitted in control reactions, where the absence of PCR-amplified DNA confirmed lack of contamination from genomic DNA. Resulting cDNA was mixed with SYBR Green PCR Master Mix (Applied Biosystems, Carlsbad, CA) for quantitative real time polymerase chain reaction (qRT-PCR) using 0.3 μM forward and reverse oligonucleotide primers (Table 1). Quantitative measures were performed using a 7300 Real Time PCR System (Applied Biosystems). Cycling conditions were an initial denaturation at 95°C for 10 min, followed by 40 cycles of 95°C for 15 seconds and 60°C for 1 minute. At the end, a dissociation curve was implemented from 60 to 95°C to validate amplicon specificity. Gene expression was calculated using absolute quantification by interpolation into a standard curve. All values were divided by the expression of the house keeping genes GAPDH or 18S.

**Table 1:** Primers used for qRT-PCR.

Target (^†^)	Sequence	Expected band size
rCIRP-F	GCATCAGATGAAGGCAAGGT	64 bp
rCIRP-R	CCAGCGCCTGCTCATTG	
rRBM3-F	TGGAGAGTCCCTGGATGGG	65 bp
rRBM3-R	TGGTTCCCCTGGCAGACTT	
mCIRP-F	GAGGCGAACAACAAGGAGAG	88 bp
mCIRP-R	GGGGAAGACCTGGTAGGAAG	
mRBM3-F	TGGTTTCATCACCTTCACCA	50 bp
mRBM3-R	CTCTCATGGCAACTGAAGCA	
mGAPDH-F	CGAGATCCCTCCAAAATCAA	205 bp
mGAPDH-R	TGACGATCTTGAGGCTGTTG	
18S (F)	ATGCTCTTAGCTGAGTGTCCCG	101 bp
18S (R)	ATTCCTAGCTGCGGTATCCAGG	

Annealing temperature for all primers was 60°C.

^†^ primers are specific for rat (r), monkey (m), or both species (18S).

### 2.4. Protein extraction and Western blotting

Additional eyes or cell lines were homogenized (1:3, w/v) in lysis buffer (20 mM HEPES, 0.2 M sucrose, 5 mM DTT, 1 mM ethylenediaminetetraacetic acid (EDTA), 10 μg/ml soybean trypsin, 10 μg/ml leupeptin, 2 μg/ml pepstatin, 0.1 mM PMSF, pH 7.4) at 4°C. Homogenates were centrifuged for 30 minutes at 15,000 x g and the supernatants collected. Protein concentration was determined by the BCA kit (Pierce, Rockford, IL), with bovine serum albumin as standard, using a NanoDrop spectrophotometer (ND100). Then, 25 μg of each sample were mixed with 4x sample buffer (Invitrogen) and heated for 10 minutes at 70°C. Samples were run on 10% SDS—polyacrylamide gels. Seeblue plus 2 Prestained Standards (Invitrogen) were used as molecular weight markers. For Western blot analysis, proteins were transferred onto 0.2-μm polyvinylidene difluoride (PVDF) membranes (iBlot system, Invitrogen). For protein identification, membranes were incubated overnight at 4°C with primary antibodies (Table 2). To standardize the results, a monoclonal IgG anti-β-actin antibody (Sigma) was used at a dilution 1:10,000 in the same membranes. To visualize immunoreactivity, membranes were incubated with anti-rabbit or anti mouse peroxidase- labeled IgGs, developed with a chemoluminiscence kit (GE Biosciences, Miami, FL), and exposed to X-ray films (GE Biosciences). Developed films were scanned with a computer-assisted densitometer (GS-800, Bio-Rad) and optical density quantified by NIH ImageJ software.

**Table 2:** Primary and secondary antibodies used in this study.

**Primary antibodies**				
**Target**	**Species**	**Dilution**	**Source**	**Reference**
CIRP	Mouse monoclonal	1:300	Proteintech	60025-2-Ig
RBM3	Rabbit monoclonal	1:1000	Abcam	ab134946
Glutamine Synthetase	Mouse monoclonal	1:300	BD Bioscience	610517
Calbindin D28K	Rabbit polyclonal	1:150	Santa Cruz Biotech.	sc-7691
Recoverin	Rabbit polyclonal	1:150	Santa Cruz Biotech.	sc-20353
**Secondary antibodies**				
**Specificity**	**Fluorochrome**	**Dilution**	**Source**	**Reference**
Donkey anti rabbit	Alexa Fluor 555	1:200	Molecular Probes	A31572
Goat anti rabbit	Alexa Fluor 488	1:200	Molecular Probes	A11008
Donkey anti mouse	Alexa Fluor 488	1:200	Molecular Probes	A21202
Goat anti mouse	Texas Red	1:200	Molecular Probes	T-862

### 2.5. Multiple immunofluorescence and confocal microscopy

Rats were deeply anaesthetized and intracardially perfused with 4% paraformaldehyde in PBS. The eyes were postfixed in the same fixative for 24 h at 4°C, cryoprotected with 20% sucrose in PBS, embedded in OCT, and sectioned in a cryostat (Leica Biosystems, Germany). Frozen sections (15 μm thick) were stained with a mixture of primary antibodies (Table 1) overnight at 4°C, exposed to the proper secondary antibodies (Table 1), and counterstained with DAPI (1:1000, Sigma). Images were acquired in a confocal microscope (TCS SP5, Leica).

## Results

### 3.1. Moderate hypothermia induces CSP expression in retinal cells in vitro

**Fig 1:**
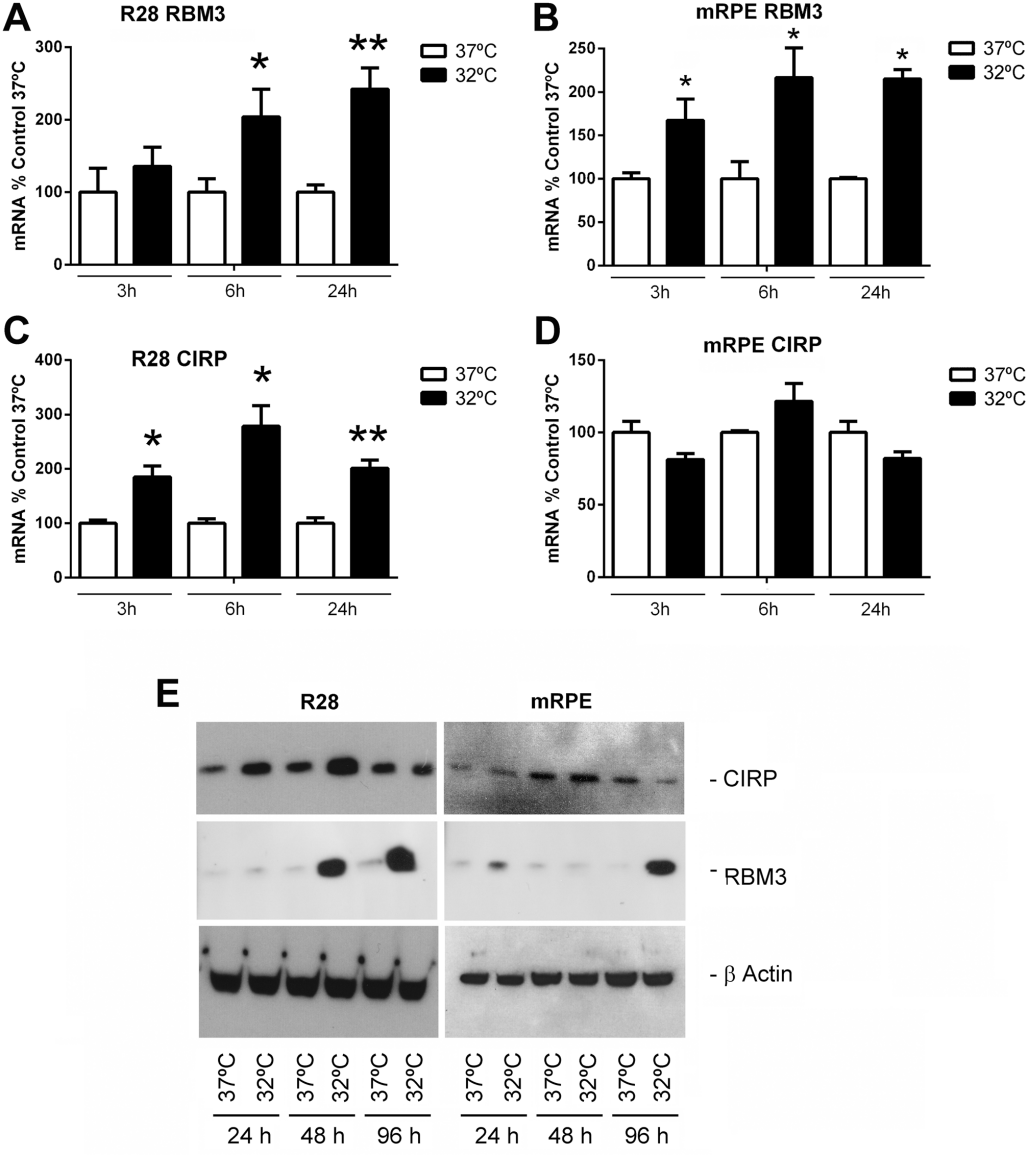
mRNA expression (A-D) and protein determination (E) for CSPs in cell lines R28 (A,C,E) and mRPE (B,D,E) at different times after cold exposure. Open bars represent mRNA expression at 37°C and closed bars at 32°C at the same times. Each bar represents the mean ± SEM of 5–8 independent measurements. Asterisks indicate statistically significant differences with cells kept in normothermia. *: p<0.05; **: p<0.01. Western blot images (E) are representative examples of 3 repeats. β–Actin was used as a loading control.

In the literature, a moderate hypothermia (32–33°C) has been described as the optimal temperature to induce CSP expression [31]. Therefore, we performed a time course with cells exposed to 32°C. RBM3 mRNA showed a clear time-dependent expression increase in R28 cells, which was statistically significant at 6h and even higher at 24h (Fig 1A). On the other hand, in mRPE cells, RBM3 was upregulated at 3h of cold exposure, and was maintained at approximately the same level up to 24h (Fig 1B). In contrast, CIRP had a differential response depending on the cell type. In R28 there was a clear overexpression of CIRP at all time points when compared with cells at 37°C (Fig 1C) but there was no significant response to moderate cold exposure in mRPE cells (Fig 1D). These mRNA results were corroborated by protein expression analyses. In R28 cells, there was a significant increase of CIRP protein in cells kept in the cold at 24h and 48h, and no change at 96h. There was also a marked increase in RBM3 protein at 48h and 96h (Fig 1E). In mRPE cells we saw no change in CIRP and a clear upregulation of RBM3 at 96h (Fig 1E).

### 3.2. Core temperature variations by exposure to environmental cold

**Fig 2:**
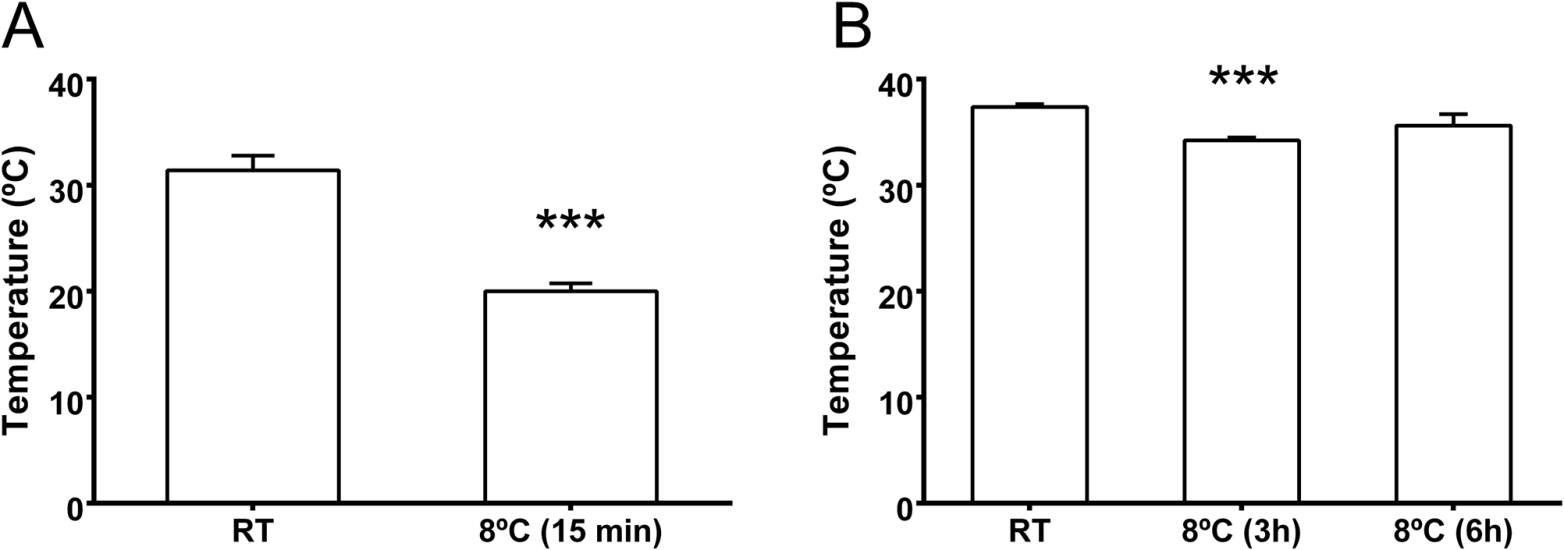
Modifications of core temperature in newborn (A) and adult (B) rats. Animals were exposed to room temperature (RT) or to a cold environment (8°C) for the indicated periods of time and their temperature was measured with a rectal probe. Each bar represents the mean ± SEM of 5–8 independent measurements. Asterisks indicate statistically significant differences with the animals kept at RT. ***: p<0.001.

Before performing in vivo experiments to localize CSPs in the retina, we studied how environmental cold influences the core temperature of neonatal and adult rats, using a rectal thermocouple. First of all, neonates had lower temperature (~31°C) than adult rats (~37°C) when kept at room temperature (Fig 2). After 15 min in a cold room at 8°C, neonates droped almost 10°C (Fig 2A). On the other hand, adult rats were more resistent to cold stress. Their core temperature droped just ~3°C after 3h in the cold room and, after that, they recovered their temperature progressively (Fig 2B).

### 3.3. CSP expression in the retina of newborn rats

**Fig 3:**
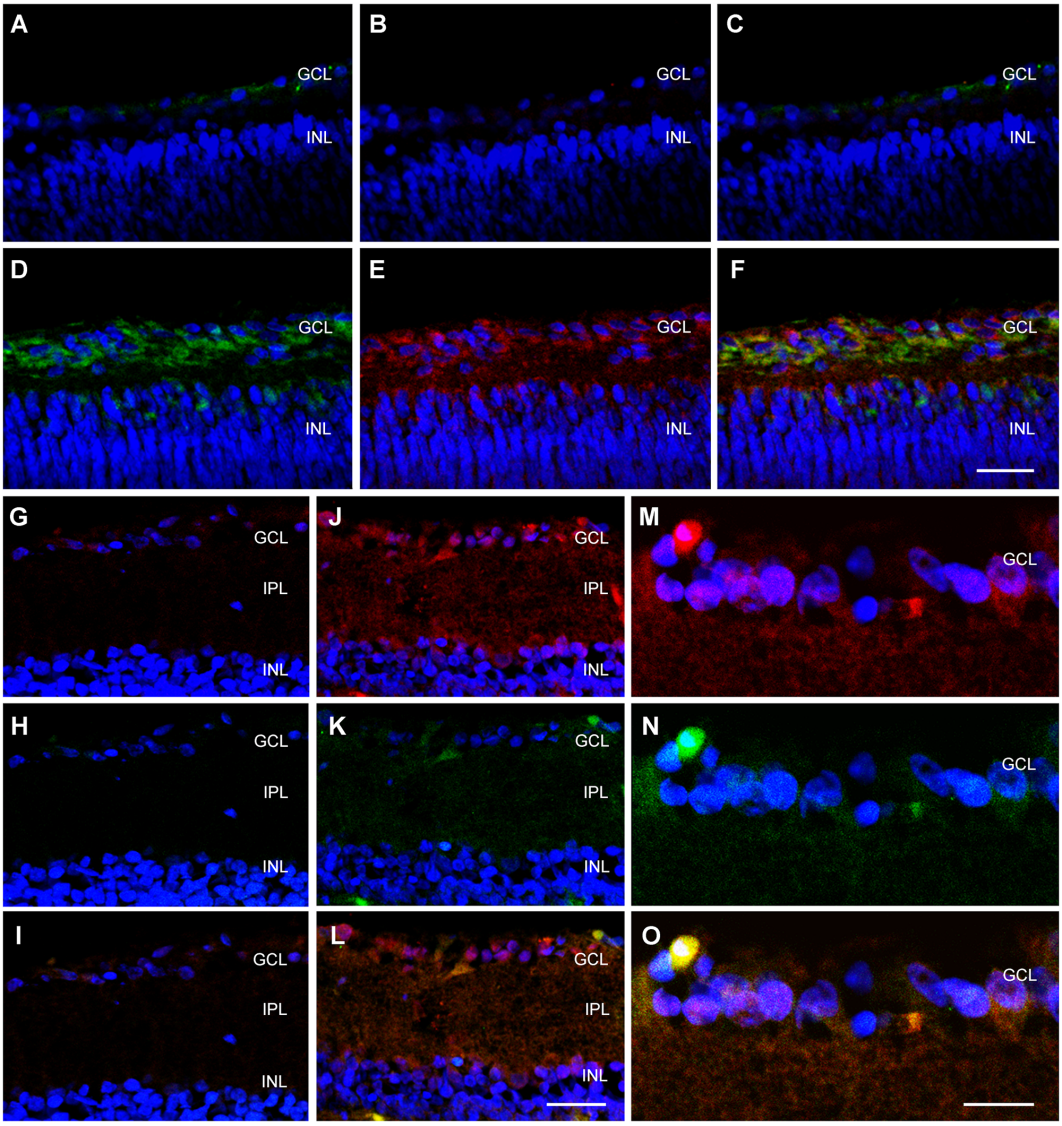
Representative confocal microscopy images of the retina of newborn (A-F) and adult (G-O) rats exposed to either room temperature (A-C, G-I) or to a cold environment (D-F, J-O), and then to room temperature for 24h before sacrifice. Sections were exposed to antibodies against RBM3 (green, A,D,H,K,N) and CIRP (red, B,E,G,H,M). An overlay of both colors can be seen at (C,F,I,L,O). GCL = ganglion cell layer, IPL = inner plexiform layer, INL = inner nuclear layer. Bar for A-L = 25 μm. Bar for M-O = 10 μm.

Newborn rats were exposed for 15 min to either normal room temperature (24°C) as a control or to 8°C, then they were kept at room temperature for 24h before being sacrificed. Sections of their retinas were stained with antibodies against RBM3 (green, Fig 3A and 3D) or CIRP (red, Fig 3B and 3E). As expected, retinas taken from animals kept at room temperature showed very little immunoreactivity for either of the CSPs (Fig 3A–3C). In animals exposed to cold temperature there was immunostaining for both proteins, and the staining was brighter in the ganglion cell layer (GCL), the inner plexiform layer (IPL), and the inner nuclear layer (INL). When signals for RBM3 and CIRP were overlayed, a certain degree of colocalization, especially in the cytoplasm of ganglion cells, was evident (Fig 3F).

### 3.4. CSP expression in the retinas of adult rats

**Fig 4:**
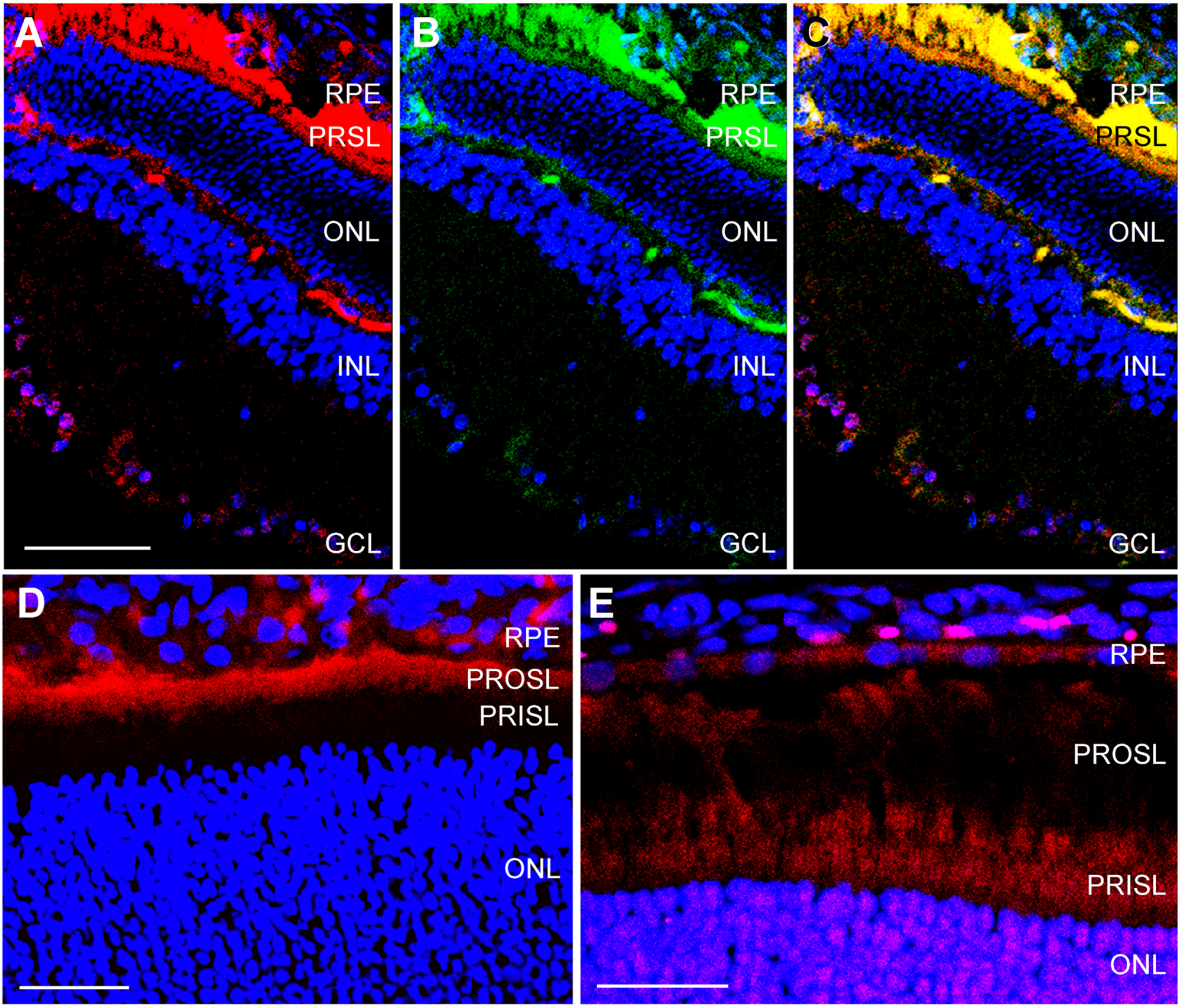
Confocal microscopy images of adult retina. Representative confocal microscopy images of the retina of adult rats exposed for 3 h to hypothermia and then for 24 h at room temperature. Sections were stained with antibodies against CIRP (red, A,D) and RBM3 (green in B, red in E). To test for colocalizations, C represents an overlay of A and B. RPE = retinal pigment epithelium, PRSL = photoreceptor segment layer, ONL = outer nuclear layer, INL = inner nuclear layer, GCL = ganglion cell layer,. Bar for A-C = 50 μm. Bar for D = 25 μm. Bar for E = 20 μm.

In the retina of adult rats kept at room temperature immunostaining for CIRP and RBM3 was negligible (Fig 3G–3I). When these animals were exposed to 3h of cold temperature, and then kept for 24h at room temperature, staining for both CSPs was evident throughout the retina (Fig 3J–3L) and colocalization of both signals was evident in particular cells (Fig 3M–3O). Immunoreactivity for both CIRP and RBM3 was found in many layers of the retina, including the RPE, photoreceptor segment layer (PRSL), outer plexiform layer (OPL), IPL, and GCL, with some expression in the nuclear layers (Fig 4A–4C). In most places, there was a clear colocalization between CIRP and RBM3 (Fig 4C). At higher magnification some interesting details surfaced. For instance, CIRP was highly expressed in the photorector outer segment layer (PROSL) but not in the photorector inner segment layer (PRISL) (Fig 4D). In contrast, RBM3 showed a stronger staining in the PRISL than in the PROSL (Fig 4E). In addition, the RPE was better defined with RBM3 (Fig 4E) than with CIRP (Fig 4D).

### 3.5. CSP expression by Western blotting

**Fig 5:**
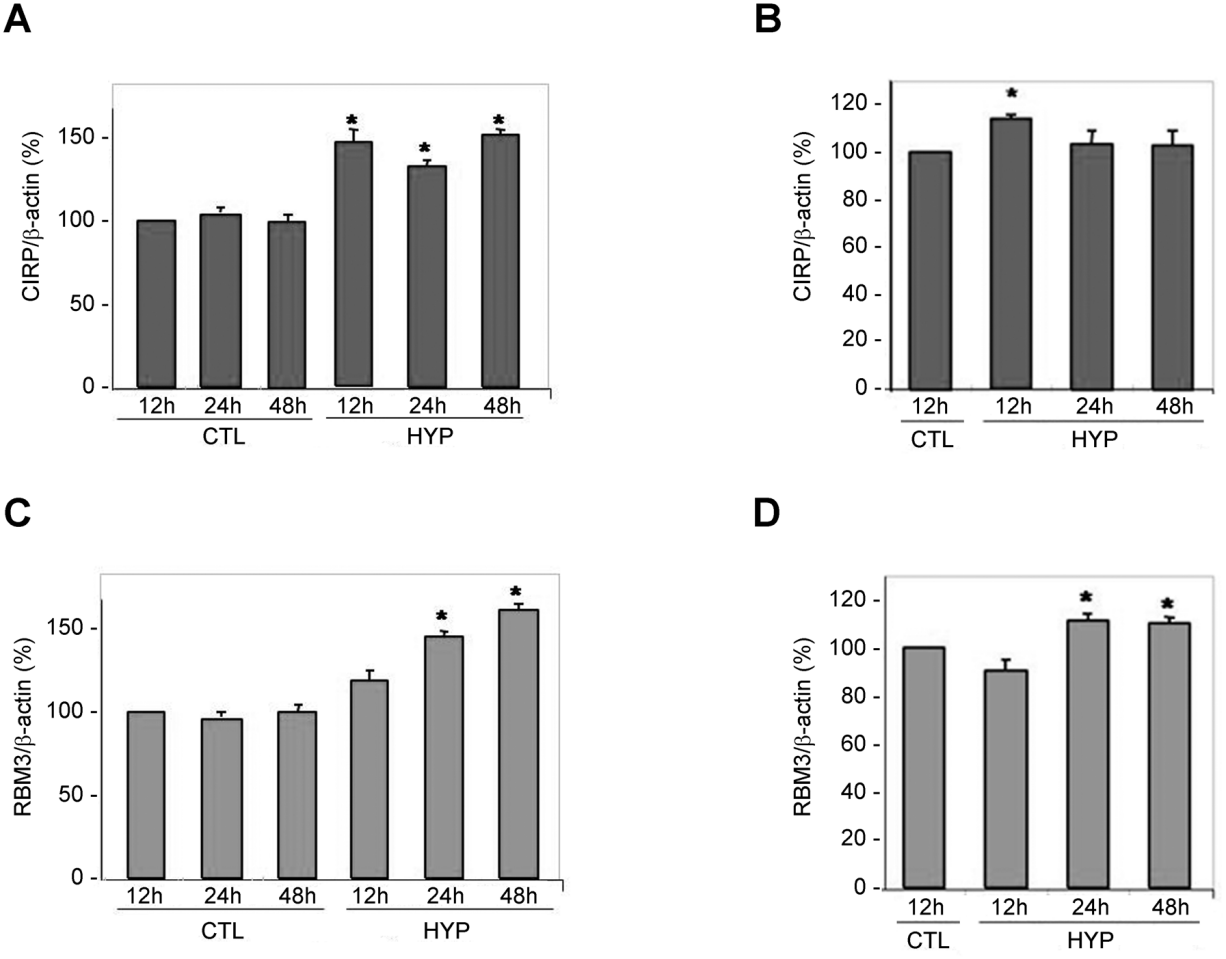
Western blot analysis of CIRP (A,B) and RBM3 (C,D) in newborn (A,C) and adult (B,D) rats. Control (CTL) animals were kept at room temperature whereas test animals were subjected to a cold environment (8°C) for 15 min (newborns) or 3 h (adults), and then sacrificed at the indicated times. Bars represent the mean ± SD of the percentage ratio of protein expression divided by the expression of β-actin for all animals (n = 6). *: p<0.05.

Eye extracts from newborn and adult rats were subjected to Western blotting with specific antibodies against CIRP and RBM3 (Fig 5). In newborn rats, CIRP expression rised significantly at 12, 24, and 48h following exposure to hypothermia for 15 min (Fig 5A). In contrast, this same protein was elevated only at 12h following hypothermia in adult rats, coming back to normal levels at 24 and 48h (Fig 5B). For RBM3, there were significant increases at 24 and 48h both for the newborns (Fig 5C) and for the adults (Fig 5D).

### 3.6. CSP expression in specific retinal cell types

**Fig 6:**
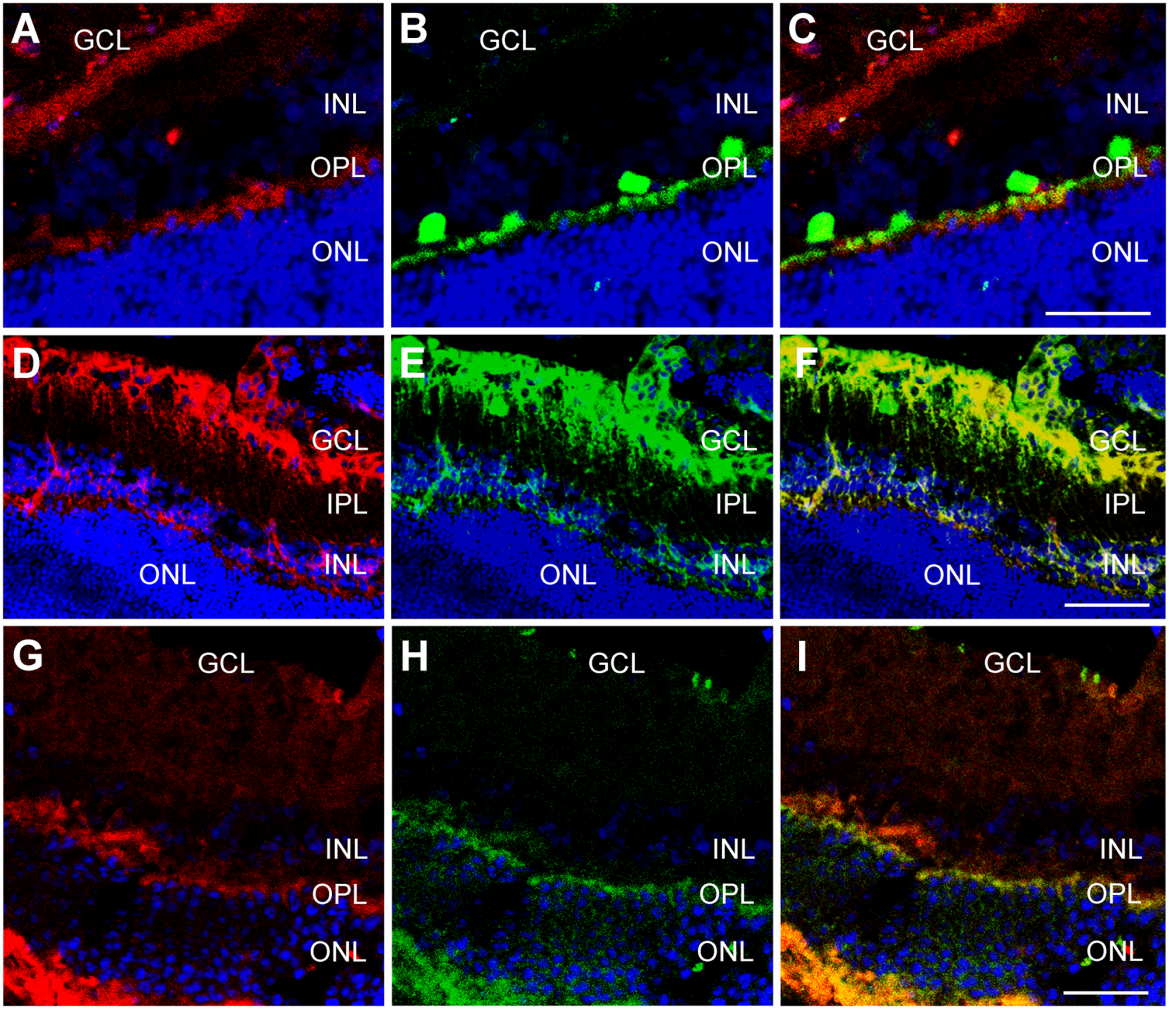
Colocalization of retinal markers with CIRP in adult retina. Representative confocal microscopy images of colocalizations in hypothermic adult rat retina between CIRP (Red, A,D,G) and cell specific markers calbindin (B), glutamine synthetase (E), and recoverin (H). The third column is a combination of the first two; a yellow hue represents colocalization. GCL = ganglion cell layer, IPL = inner plexiform layer, INL = inner nuclear layer, OPL = outer plexiform layer, ONL = outer nuclear layer. Bar for A-C = 50 μm. Bar for D-F = 100 μm. Bar for G-I = 50 μm.

To better understand the distribution of the immunoreactivity for both CSPs, colocalization studies were performed. We used cell markers as described [35,36], employing calbindin to label horizontal cells, glutamine synthetase for Müller cells, and recoverin for cone bipolar cells and photoreceptors. CIRP was found in the cell processes of horizontal cells but not in their somas (Fig 6A–6C). There was a complete colocalization of CIRP with the inner process and cytoplasmic marker of Müller cells (Fig 6D–6F). Also a complete colocalization of CIRP with recoverin indicated the presence of this CSP in the cytoplasm of cone bipolar cells in the ONL and OPL and also in the inner and outer segments of the photoreceptor layer (Fig 6G–6I).

**Fig 7:**
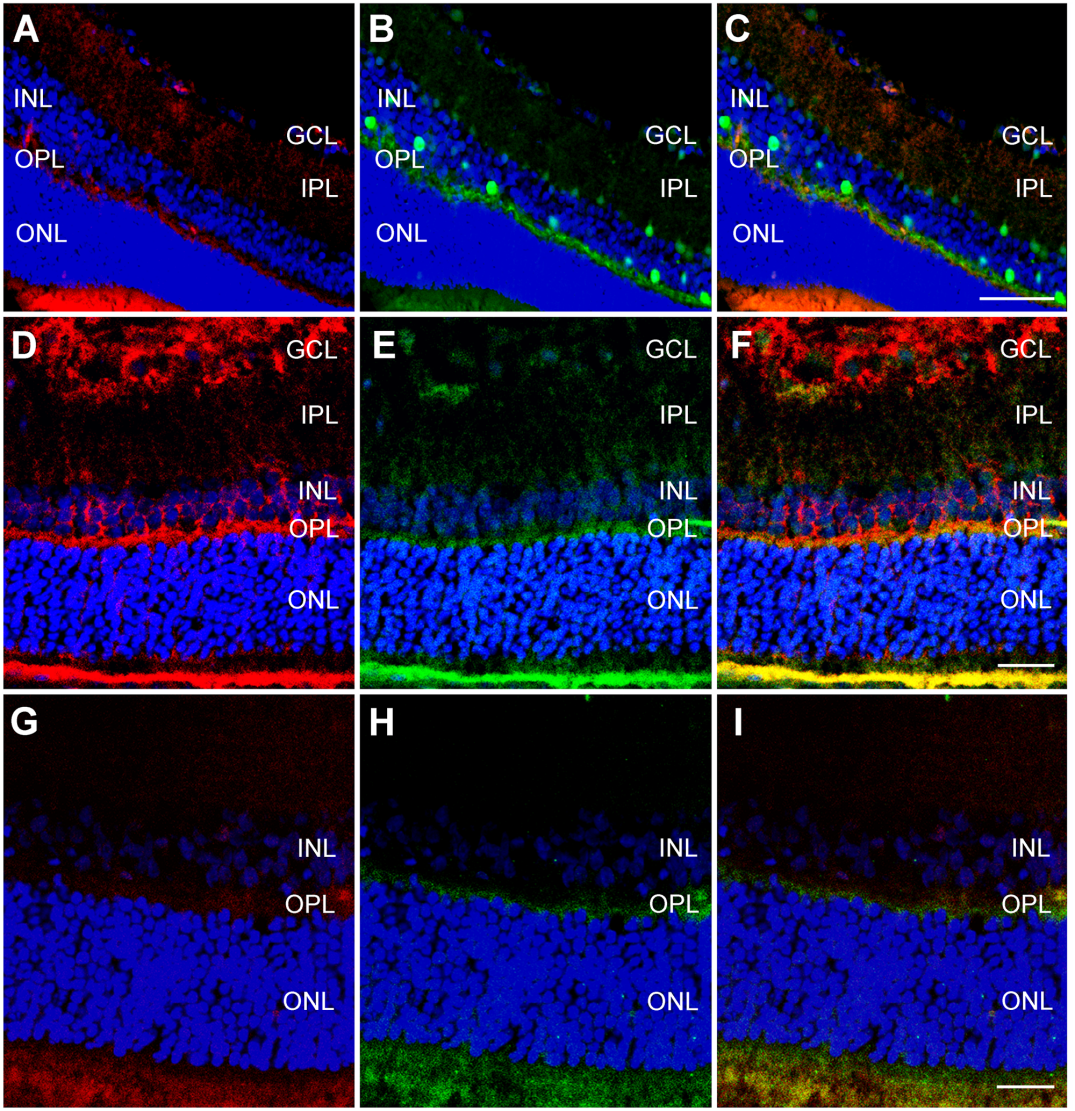
Colocalization of retinal markers with RBM3 in adult retina. Representative confocal microscopy images of colocalizations in hypothermic adult rat retina between RBM3 (A,E,G) and cell specific markers calbindin (B), glutamine synthetase (D), and recoverin (H). The third column is a combination of the first two; a yellow hue represents colocalization. GCL = ganglion cell layer, IPL = inner plexiform layer, INL = inner nuclear layer, OPL = outer plexiform layer, ONL = outer nuclear layer. Bar for A-C = 50 μm. Bar for D-F = 25 μm. Bar for G-I = 25 μm.

RBM3 had a similar distribution to CIRP, being present in the cell processes of horizontal cells (Fig 7A–7C), the cytoplasm of Müller cells (Fig 7D–7F), and of the cone bipolar cells and photoreceptors (Fig 7G–7I).

## Discussion

In this study we have shown that the expression for both CIRP and RBM3 is activated upon exposure to a cold environment in retinal cell lines and in the retina of neonatal and adult rats.

Using retina-derived cell lines we found a time-dependent increase of RBM3 in both R28 and mRPE. On the other hand, CIRP was elevated by cold in R28 but not in mRPE. This was also confirmed by Western blotting. Interestingly, looking at adult rat retina exposed to cold, we found a nice expression of RBM3 in the RPE whereas staining for CIRP was inconclusive. This suggests that at least CIRP is regulated in a cell type-dependent fashion, which justifies an in-depth anatomical study.

Before performing in vivo experiments, we studied the influence of external cold environments in the temperature of the animals. It has been reported that the most important determinant of effective cooling is body surface area. In addition, age is also important with very young and older individuals being impaired for effective temperature regulation [37]. It has been published that the eye, and specially the retina, has an active defense mechanism against cold, based on the modulation of choroidal blood flow [38]. Obviously this mechanism of defense would depend on the size of the eye and also on the maturity of the eye anatomy. In our study, the temperature drop was very quick and very pronounced in newborn rats whereas in adults we were able to obtain only a small reduction in internal temperature. This fact may be a limitation for the possibilities of applying therapeutic hypothermia to the eye from an external source. In fact, in the case of brain therapeutic hypothermia, this treatment must be applied through external cooling of the blood flowing through the central nervous system [1,4].

We found a clear upregulation of both CSPs in the retina of neonatal rats, specially in the region that would become the GCL. This neat increase in expression may be part of the molecular mechanism underlying the beneficial effects of hypothermia in the retina when applied to experimental neonates subjected to hypoxia/asphyxia [15–17]. In a recent clinical trial, neonates with hipoxic-ischemic encephalopathy were subjected to head cooling combined with whole-body cooling to 33.5°C for 72 h and the results showed that therapeutic hypothermia resulted in decreased brain tissue injury, improved survival, and better neurological outcomes [39,40]. It would be interesting to test the efficacy of such applications to retinal outcomes.

Immunoreactivity for both CSPs was also found in adult rat retinas exposed to cold. In this case we found a higher degree of colocalization between CIRP and RBM3 than in neonates. Interestingly, there was differential expression of these two proteins in the PRSL with CIRP being expressed exclusively in the outer segment and RBM3 mostly in the inner segment. Both segments are connected by specialized cilia and the outer segment houses the rhodopsin discs. In theory, not many mRNAs should be located in the outer segment of the photoreceptors, but some of them have been localized there [41] and CIRP may have a function in regulating the expression of such mRNAs.

Specificity of the CIRP and RBM3 staining was demonstrated by the fact that the retina of rats kept at room temperature had a negligible staining for either protein, and that the immunoreactivity was intense following cold exposure, as expected from working CSPs. Interestingly, Western blots of the eye showed a higher expression of these proteins in normothermic animals than expected from the immunostaining images. Perhaps this higher expression is due to additional eye structures, other than the retina. This possibility will be explored in future studies.

Both CSPs have been localized in the cytoplasm of several retinal cell types following cold stress. These cell types included ganglion cells, Müller cells, horizontal cells, cone bipolar cells, photoreceptors, and RPE. This extensive localization suggests that CSPs exert a profound impact in the physiology of the mammalian retina when exposed to hypothermia. These results may explain the protective effects that exposure to cold temperatures has on ischemic retinas [11–14]. All the physiological effects of CSPs are thought to be mediated by their binding to cellular mRNAs and the concomitant modification of their half life [24,42]. Several studies have tried to determine the RNA minimal motif needed for CSP binding. A paper on the influence of CSPs in the testis proposed a binding motif as simple as UUU [42], with the consequence that about half of the genes of the genome possess such sequence and therefore should be potential targets for CSPs. Other studies propose more complex motifs [24] but still the number of potential mRNA targets is very high. Future studies should identify which particular mRNAs are modulated by CSPs in the retina and, by doing so, try to dissect the exact mechanism by which hypothermia and CSPs contribute to cell preservation in the retina.

## Conclusions

CSP expression rapidly rises in the mammalian retina following exposure to hypothermia in a cell type-specific pattern. This observation may be at the basis of the molecular mechanism by which hypothermia exerts its therapeutic effects in the retina.

## Abbreviations

CIRP or CIRBP: Cold inducible RNA-binding protein
CSP: Cold-shock protein
GCL: Ganglion cell layer
INL: Inner nuclear layer
IPL: Inner plexiform layer
ONL: Outer nuclear layer
OPL: Outer plexiform layer
PRISL: Photoreceptor inner segment layer
PROSL: Photoreceptor outer segment layer
PRSL: Photoreceptor segment layer
qRT-PCR: Quantitative real time polymerase chain reaction
RBM3: RNA-binding motif protein 3
RPE: Retinal pigment epithelium
VEGF: Vascular endothelial growth factor
